# Point‐of‐care ultrasound‐guided pericapsular nerve group block for superior pubic ramus fracture in the emergency department: A case report

**DOI:** 10.1002/ajum.12308

**Published:** 2022-07-03

**Authors:** Elinor Cripps, Alan Fahey, Peter James Snelling

**Affiliations:** ^1^ Lismore Base Hospital Lismore New South Wales Australia; ^2^ Emergency Department Gold Coast University Hospital Southport Queensland Australia; ^3^ School of Medicine and Dentistry Griffith University Southport Queensland Australia; ^4^ Sonography Innovation and Research (Sonar) Group Southport Queensland Australia; ^5^ Child Health Research Centre University of Queensland Southport Queensland Australia

**Keywords:** analgesia, emergency medicine, nerve block, pelvic fractures, pericapsular nerve group block, pubic bone, pubic ramus, regional anaesthesia

## Abstract

Pelvic fragility fractures, such as pubic ramus fractures, are a common and painful condition in the elderly population. Despite this, there are few regional anaesthesia options available to effectively relieve pain in these fracture types and avoid potential side effects from opioid administration. This case report describes an elderly patient with a superior ramus fracture, who received effective pain relief with motor sparing, using a standard‐volume point‐of‐care ultrasound‐guided pericapsular nerve group (PENG) block performed in the emergency department. The standard‐volume PENG block performed by an emergency clinician appears to be a safe, effective and feasible regional anaesthesia technique for superior pubic ramus fracture, with the additional benefit of motor sparing that may potentially facilitate earlier mobilisation and discharge.

## Introduction

Pelvic fragility fractures in the elderly population, such as pubic ramus fractures, are a common presentation to the emergency department (ED).^1^ The pain associated with these fractures can be notoriously difficult to manage but is generally conservatively managed with the goal of early mobilisation.^2^ Regional anaesthesia is frequently used for proximal femur fractures as an effective adjuct to reduce opioid analgesic administration, but currently, there is no equivalent routinely used anaesthesia technique for pubic rami fractures.

The point of care ultrasound (POCUS) guided pericapsular nerve group (PENG) block is an emerging regional anaesthesia technique that may be safely used to provide effective analgesia for these patients in the ED.^3^, ^4^ The PENG block, a variation of the iliopsoas plane block, was originally designed to improve perioperative analgesia in hip fracture.^5^ This single‐shot block theoretically targets the articular branches of the femoral, obturator and accessory obturator nerve within the iliopsoas compartment, providing extensive coverage of the hip capsule with potential motor sparing,^6^ although the obturator nerve branches may only be variably anaesthetised.^7^

We report the first case of the standard‐volume POCUS‐guided PENG block for acute pain in superior pubic ramus fracture with motor sparing. This may provide additional benefits such as avoidance of opioid‐related side effects and early mobilisation to facilitate discharge. Consent to publish the case report was obtained from the patient and approved by the North Coast New South Wales Human Research and Ethics Committee (EC00415).

## Case study

An 82‐year‐old woman was brought to the ED with right hip pain following an unwitnessed fall. A PENG block was performed due to the suspicion of neck‐of‐femur fracture on the X‐ray. Subsequent CT imaging confirmed an isolated right superior pubic ramus fracture.

**Figure 1:**
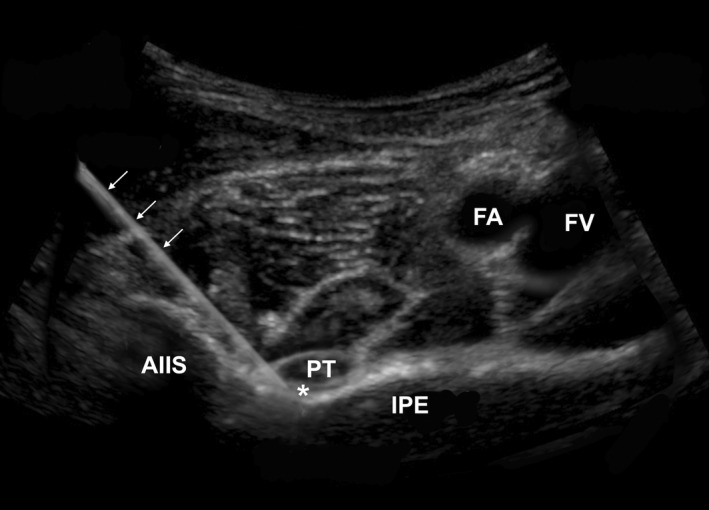
Ultrasound view of the pericapsular nerve group block showing anatomical landmarks, with superimposed needle (arrows) at the target site (*). FA, femoral artery; FV, femoral nerve; AIIS, anterior–inferior iliac spine; PT, psoas tendon; IPE, iliopubic eminence.

The PENG block was performed using the POCUS guidance by an emergency clinician under aseptic conditions. With the patient in a supine position, a curvilinear probe (C60xp 5‐2 MHz; Fujifilm Sonosite Xporte, Bothell, Washington, USA) was placed in the inguinal crease, with 45° medial rotation. The probe was moved cephalad to visualise the anterior–inferior iliac spine (AIIS), the iliopubic eminence (IPE) and the psoas tendon (Figure 1). A small volume of 1% lignocaine was infiltrated superficially to anaesthetise the skin. A 22‐gauge 80‐mm needle (SonoBlock; PAJUNK®, Geisingen, Baden‐Württemberg, Germany) was advanced lateral to medial at 30–45°. The needle was guided along the AIIS towards the bony surface of the ilium between the AIIS and the IPE and advanced to a bony end point posterior to the psoas tendon (Figure 1). 20 mL of 0.375% ropivacaine was injected, with the psoas tendon and muscle seen to hydro‐dissect off the ilium. No complications were observed.

Immediately prior to block, the patient's visual analogue scale pain score was 7, reducing to a score of 1 at 13 min post‐block. The patient performed a straight leg raise at this time. The patient only received immediate‐release 5 mg oxycodone orally 3 h prior to the block. The patient subsequently had no requirements for opioid analgesia for 24 h and consistently scored a pain score of 0–1. The patient mobilised to baseline and was discharged the following morning.

## Discussion

Regional anaesthesia options for pelvic fractures, such as pubic ramus fractures, are neuraxial and lumbar plexus blocks. These complex techniques are not feasible or safe to routinely perform in the ED. The PENG block is emerging as a suitable alternative in the ED for this common yet neglected fracture.^3^ However, it remains unclear how the PENG block anaesthetises the superior pubic ramus, given the distance between block and target site, and that the medial spread of injectate appears to be limited by iliac fascia.^5^, ^6^, ^7^

The specific sensory innervation of the superior and inferior pubic rami is not known,^8^ making it difficult to conceptualise the mechanism of action of the PENG block. Hypotheses include the blockade of relevant sensory branches within the iliopsoas plane, direct spread along periosteum to create a haematoma block,^3^ medial spread to pectineus muscle to block sensory branches of the obturator nerve,^6^ superior spread across iliopectineal fascia to block more proximal obturator and femoral nerve branches.^9^ Additional anatomical studies are required to further characterise the sensory innervation of the pubic rami and the mechanism of the PENG block.

The ideal injectate volume for the PENG block has not yet been elucidated.^10^ The greater the volume is injected, the more likely the injectate will capture the high branches of the femoral nerve, potentially leading to quadriceps weakness.^11^ On the contrary, a lower injectate volume may reduce sensory blockade overall. The earliest successful use of the high‐volume (30 mL) PENG block was reported in three patients with pelvic fragility fractures, but motor function preservation was not assessed.^3^ Motor function preservation is an important component of the PENG block for stable pelvic fractures, to reduce fall risk and allow for early mobilisation, which may potentially facilitate timely discharge.

In this case report, effective analgesia was achieved with motor‐sparing effects using the standard‐volume PENG block in the ED for an elderly patient with a superior pubic ramus fracture. Performing a PENG block in this patient group may particularly have a role in cases where oral analgesia is inadequate and allow early mobilisation. Prospective studies are required to determine the safety, feasibility and effectiveness of the PENG block, including the ideal injectate volume, for elderly patients with pubic ramus fractures in the ED.

## Funding

No funding information is provided.

## Conflict of interest

No conflict of interest is declared.
